# High expression of miR-17-5p in tumor epithelium is a predictor for poor prognosis for prostate cancer patients

**DOI:** 10.1038/s41598-021-93208-6

**Published:** 2021-07-05

**Authors:** Maria Jenvin Stoen, S. Andersen, M. Rakaee, M. I. Pedersen, L. M. Ingebriktsen, R. M. Bremnes, T. Donnem, A. P. G. Lombardi, T. K. Kilvaer, L. T. Busund, E. Richardsen

**Affiliations:** 1 Translational Cancer Research Group, Institute of Medical Biology, UiT the Arctic University of Norway, 9037 Tromso, Norway; 2 Translational Cancer Research Group, Institute of Clinical Medicine, UiT the Arctic University of Norway, Tromso, Norway; 3 Department of Clinical Pathology, University Hospital of North Norway, Tromso, Norway; 4 Department of Oncology, University Hospital of North Norway, Tromso, Norway; 5 Centre for Cancer Biomarkers CCBIO, Department of Clinical Medicine, Section for Pathology, University of Bergen, 5021 Bergen, Norway

**Keywords:** Prognostic markers, miRNAs, Prostate cancer, Tumour biomarkers

## Abstract

MicroRNAs (miRs) are small non-coding RNA molecules, which are involved in the development of various malignancies, including prostate cancer (PCa). miR-17-5p is considered the most prominent member of the miR-17-92 cluster, with an essential regulatory function of fundamental cellular processes. In many malignancies, up-regulation of miR-17-5p is associated with worse outcome. In PCa, miR-17-5p has been reported to increase cell proliferation and the risk of metastasis. In this study, prostatectomy specimens from 535 patients were collected. Tissue microarrays were constructed and in situ hybridization was performed, followed by scoring of miR-17-5p expression on different tumor compartments. High expression of miR-17-5p in tumor epithelium was associated with biochemical failure (BF, *p* < 0.001) and clinical failure (CF, *p* = 0.019). In multivariate analyses, high miR-17-5p expression in tumor epithelial cells was an independent negative prognostic factor for BF (HR 1.87, 95% CI 1.32–2.67, *p* < 0.001). In vitro analyses confirmed association between overexpression of miR-17-5p and proliferation, migration and invasion in prostate cancer cell lines (PC3 and DU145). In conclusion, our study suggests that a high cancer cell expression of miR-17-5p was an independent negative prognostic factor in PCa.

## Introduction

Prostate cancer (PCa) is the most common cancer among men in developed countries^1^. In 2018, prostate cancer was responsible for 7.1% of all cancers in men, and 3.8% of all cancer deaths in men^2^. Considering the low sensitivity and specificity of serum Prostate Specific Antigen (PSA) levels, there is a need for better PCa biomarkers^3^.

MicroRNAs (miRs) are small non-coding RNA molecules, with 18–25 nucleotides in length^4^, which regulate gene expression post-transcriptionally^5^. miRs regulate important cell-function such as proliferation, differentiation, metabolism, cell cycle, stem cell maintenance and apoptosis^3^. Dysfunctional miRs are associated with the development of several diseases, including cancer^6^. In cancer, miRs are categorized as oncogenic or tumor-suppressive^6^. miR expression may be specific for different cancer types, stages and metastasis, making them potential diagnostic, prognostic and predictive biomarkers^3, 7^. miRs are especially attractive in a biomarker setting because they are stable and readily available in blood and other body-fluids as well as in tissue^8–10^.

The miR-17-92 cluster is located on the non-protein-coding gene MIR17HG (the miR-17-92 cluster host gene) on chromosome 13^11^. It transcribes the six mature miRs; miR-17, miR-18a, miR-19a, miR-19b, miR-20a and miR-92a^11^. These miRs are known for regulating the genes related to cell cycle, angiogenesis and apoptosis pathways^12^. The miR-17-92 gene cluster plays an oncogenic role in various malignancies^11^, including PCa^13^. Its oncogenic activity is stimulated through activation of PI3K/AKT/mTOR and JAK-STAT, and suppression of PTEN and SOCS-1 pathways^11^. The six miRs are grouped into four miR families, of which the miR-17 family (miR-17 and miR-20a) is one group^12^.

**Figure 1:**
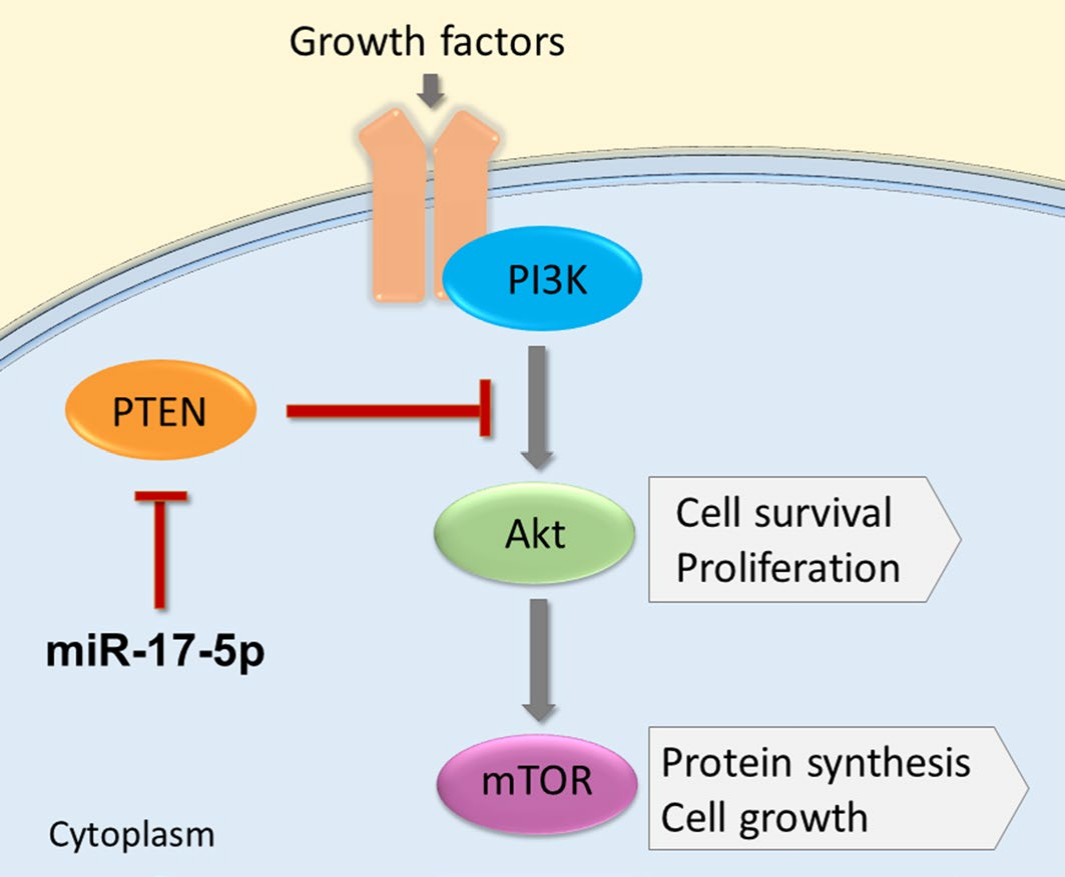
miR-17-5p inhibitorial mechanism on the PI3K/AKT/mTOR pathway in PCa. A simplified illustration of the PI3K/AKT/mTOR pathway, and miR-17-5p upregulation through inhibition of the tumor suppressor PTEN.

miR-17-5p is the most prominent member of the miR-17-92 cluster^14^. It is essential for fundamental cellular processes like proliferation, cell cycle regulation and apoptosis^14^. In PCa, miR-17-5p is reported to have oncogenic potential, important for cancer development and invasion^15^. It inhibits the tumor suppressors TIMP3, PTEN (Fig. 1) and p21, resulting in tumor development via increased cell cycling, proliferation and tumor metastasis^15^. miR-17-5p is overexpressed in PCa compared to benign prostate tissues^16^. A high expression of miR-17-5p is associated with biochemical recurrence and an aggressive PCa phenotype^17^. Hence, dysregulated miR-17-5p in serum has been suggested as potential predictive biomarkers in PCa^17^. However, the role of the miR-17-92 cluster in PCa progression is still poorly understood^18^.

Previously, our group have conducted two smaller micro-array screening studies in prostate cancer tissue and in breast cancer tissue *vs* benign controls. In the prostate cancer study, miR-17-5p was not found to be significantly up- or downregulated^19^. Whereas, in the breast cancer study, miR-17-5p was found to be expressed in higher levels in high grade breast cancer tumors^20^. Herein, we have thoroughly investigated the miR-17-5p expression in tumor epithelium and tumor-associated stromal areas in human prostate glands, and evaluated its prognostic impact in a large PCa cohort.

## Results

### Patient characteristics

Supplementary Table S1 presents an overview of clinicopathological characteristics for the 535 patients included in this study. Based on the last follow-up data (31/12/2015), 200 patients (37%) experienced biochemical failure (BF), 56 patients (11%) experienced clinical failure (CF) and 18 patients (3.4%) had died of prostate cancer (PCD). The median serum PSA level was 8.8 ng/ml (range 0.7–104 ng/ml). The median age at prostatectomy surgery was 62 years (range 47–75 years) and median tumor size was 20 mm (range 2–50 mm).

### MicroRNA expression and cut-off

**Figure 2:**
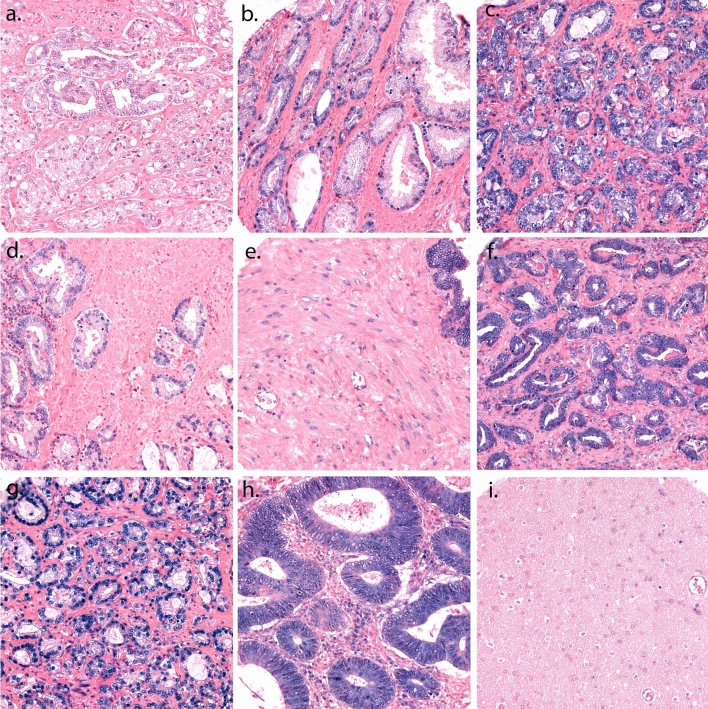
A panel of representative cores with scoring of tumor epithelium and tumor stroma for miR-17-5p stained by ISH. TE is scored by the intensity of the staining in the cell cytoplasm and core, with values from 0–3, and TS is scored by the density of positive cells in the examined core. (**a**) Score 1 in TE; (**b**) Score 2 in TE; (**c**) Score 3 in TE; (**d**) Score 1 in TS; (**e**) Score 2 in TS; (**f**) Score 3 in TS; (**g**) U6 control staining; (**h**) Positive tissue control: normal human colon tissue; (**i**) Negative tissue control: normal human brain tissue. Magnification 20x.

Figure 2 represents different levels of miR-17-5p expression on the scoring of tumor epithelium (TE) and tumor stroma (TS). TE was scored by the intensity of the staining, with values from 0–3 (0 = negative, 1 = weak, 2 = moderate and 3 = strong). TS was scored based on density of positive cells in the examined core using following scales: 1 = 1–20%, 2 = 21–50% and 3 > 50%. High miR-17-5p expression in TE and TS was defined as a score ≥ 2.5 and ≥ 1.5 respectively. The median scoring value was used for all cut-off values, and the scoring values were dichotomized into the categories high and low expression. The TE + TS variable was created by adding the dichotomized values from TE and TS together, creating a variable with 3 categories (low/low expression = 0, mixed expression = 1, and high/high expression = 2). 402 patients had cores available for scoring for miR-17-5p in TE (133 missing patients), and 469 patients were available for scoring in TS (66 missing patients). A patient was categorized missing if the patient’s cores was fallen off from the slide during staining, or if there were no visible malignant cells in the cores. High intraclass correlation was achieved between the uro-pathologist (ER) and the trained investigators (LMI/MJS) for miR-17-5p in TE (ICC: 0.94, *p* < 0.001) and TS (ICC: 0.88, *p* < 0.001)**.**

### miR-17-5p correlations with clinicopathological variables

Supplementary Table S2 presents the significant associations between miR-17-5p expression and clinicopathological variables. There was a relationship between positive apical margin (PAM) and a high miR-17-5p expression in TE, TS and TE + TS (*p* = 0.022, *p* = 0.001 and *p* = 0.004, respectively) and perineural infiltration and TE, TS and TE + TS (*p* < 0.001, *p* < 0.001 and *p* < 0.001, respectively). An association between pT-stage (2, 3a or 3b) with TS (*p* = 0.026) and TE + TS (*p* = 0.030) was seen, as well as vascular infiltration and high TS (*p* = 0.015) and perineal prostatectomy and high TS and TE + TS (*p* = 0.039 and *p* = 0.014, respectively). TE and TS showed a significant correlation with each other (*p* < 0.001).

### Univariate analysis

**Figure 3:**
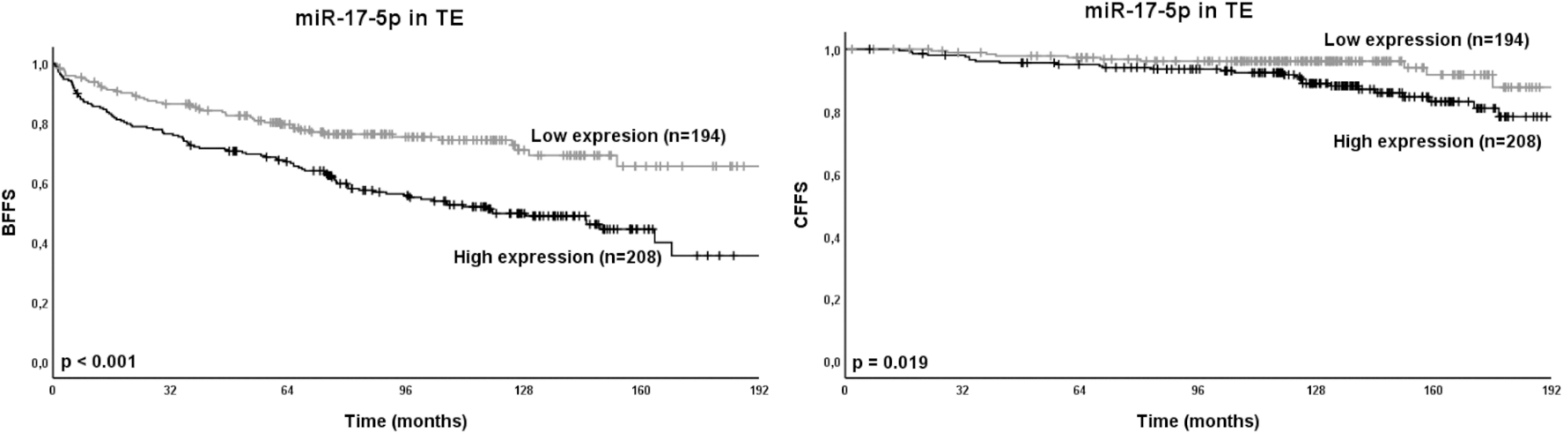
Relation between miR-17-5p in TE and the prostate cancer outcomes BFFS and CFFS presented in Kaplan–Meier curves. miR-17-5p was divided into low and high expression for the 402 available cores. Abbreviations: BFFS = biochemical failure-free survival; CFFS = clinical failure-free survival; p = p-value; TE = tumor epithelium.

Results for the univariate analyses and clinicopathological variables are presented in Supplementary Table S1. The univariate analyses for miR-17-5p and BFFS, CFFS and PCDFS are presented in Table 1. High expression of miR-17-5p (TE: *p* < 0.001, TS: *p* = 0.007, TE + TS: *p* < 0.001) were significantly associated with BF. Further, high expression of miR-17-5p in TE (*p* = 0.019) were significantly associated with CF. Figure 3 presents Kaplan–Meier survival curves for miR-17-5p in TE compared to BFFS and CFFS, and Supplementary Figure S1 presents Kaplan–Meier curves for all significant univariate outcomes.

**Table 1:** Expression of miR-17-5p as predictor for BFFS, CFFS and PCDFS in prostate cancer patients. Univariate analysis; log rank.

miR-17-5p expression		Patients		BFFS			CFFS			PCDFS		
		n	%	5-year (%)	10-year (%)	*p*	5-year (%)	10-year (%)	*p*	5-year (%)	10-year (%)	*p*
**TE**	Low	194	36.3	80	74	< 0.001*	98	96	**0.019**	99	98	0.345
	High	208	38.9	69	50		95	92		99	97	
	Missing	133	24.9									
**TS**	Low	188	35.1	81	71	0.007*	98	97	0.050	99	98	0.559
	High	281	52.5	72	57		96	93		100	98	
	Missing	66	12.3									
**TE + TS**	Low/low	107	20.0	83	75	< 0.001*	98	98	0.053	99	99	0.404
	Mixed	124	23.2	77	67		97	94		99	97	
	High/high	170	31.8	67	48		95	91		99	98	
	Missing	134	25.0									

Significant p-values marked in bold for p < 0.05, and * for p < 0.01. Abbreviations: BFFS = biochemical failure-free survival; CFFS = clinical failure-free survival; p = p-value; PCDFS = prostate cancer death-free survival; TE = tumor epithelium; TS = tumor stroma.

### Multivariate analysis

Results from multivariate analyses are presented in Table 2. High miR-17-5p expression in TE and TE + TS were independently associated with BF (HR 1.87, 95% CI 1.32–2.67, *p* < 0.001 and HR (high expression) = 1.81, 95% CI 1.15–2.85, respectively). In addition, the following clinicopathological variables were significant independent predictors for BF: Preoperative PSA, tumor size, pT-stage and perineural infiltration. For CF, ISUP Grade group and preoperative PSA were independent prognostic factors. CF with TS and TE + TS was borderline significant (*p* = 0.05 and *p* = 0.053, respectively) in the univariate analysis. These are interesting trends, and therefore included in the multivariate model. In the multivariate analysis for CF, neither TE, TS nor TE + TS was significant and therefore CF is not shown in Table 2. miR-17-5p expression in TE, TS and TE + TS were not significant in univariate analysis for PCDFS, and hence not entered into multivariate analyses.

**Table 2:** Expression of miR-17-5p and significant clinicopathological variables independent prognostic impact on BF (Cox regression analyses, n = 535).

Characteristic	BF (200 events)			
	Model 1		Model 2	
	HR (95% CI)	*p*	HR (95% CI)	*p*
**ISUP Grade Group**	NS		NS	
**Preop. PSA**		< 0.001		< 0.001
PSA < 10	1		1	
PSA > 10	1.94 (1.39–2.73)		1.88 (1.34–2.64)	
**Tumor Size**		0.042		0.030
≤ 20 mm	1		1	
> 20 mm	1.45 (1.01–2.08)		1.49 (1.04–2.14)	
**pT-stage**		< 0.001		< 0.001
pT2	1		1	
pT3a	1.63 (1.10–2.43)	0.016	1.61 (1.08–2.40)	0.019
pT3b	3.41 (2.14–5.42)	< 0.001	3.39 (2.12–5.42)	< 0.001
**pN-stage**	NE		NE	
**PNI**		0.014		0.026
No	1		1	
Yes	1.58 (1.10–2.27)		1.53 (1.05–2.21)	
**LVI**	NS		NS	
**Margin**	NS		NS	
**PCM**	NS		NS	
miR-17-5p in TE*		< 0.001	NE	
Low expression	1			
High expression	1.87 (1.32–2.67)			
miR-17-5p in TS*	NS		NE	
miR-17-5p in TE + TS*	NE			0.004
Low/low expression			1	
Mixed expression			1.03 (0.62–1.70)	0.916
High/high expression			1.81 (1.15–2.85)	0.011

Multivariate analysis with *p* ≤ 0.05 from the univariate analyses (Table 1 and Supplementary Table S1).

*The variables TE and TS was included in the same model, while the TE + TS variable was in a separate model.

Abbreviations: BF = biochemical failure; CF = clinical failure; LVI = lympho-vascular infiltration; NE = not entered; NS = not significant; p = p-value; PAM = positive apical margin; PCM = positive circumferent margin; PNI = perineural infiltration; Preop = preoperative; PSA = prostate specific antigen; TE = tumor epithelium; TS = tumor stroma.

### miR-17-5p overexpression in PCa cell lines

**Figure 4:**
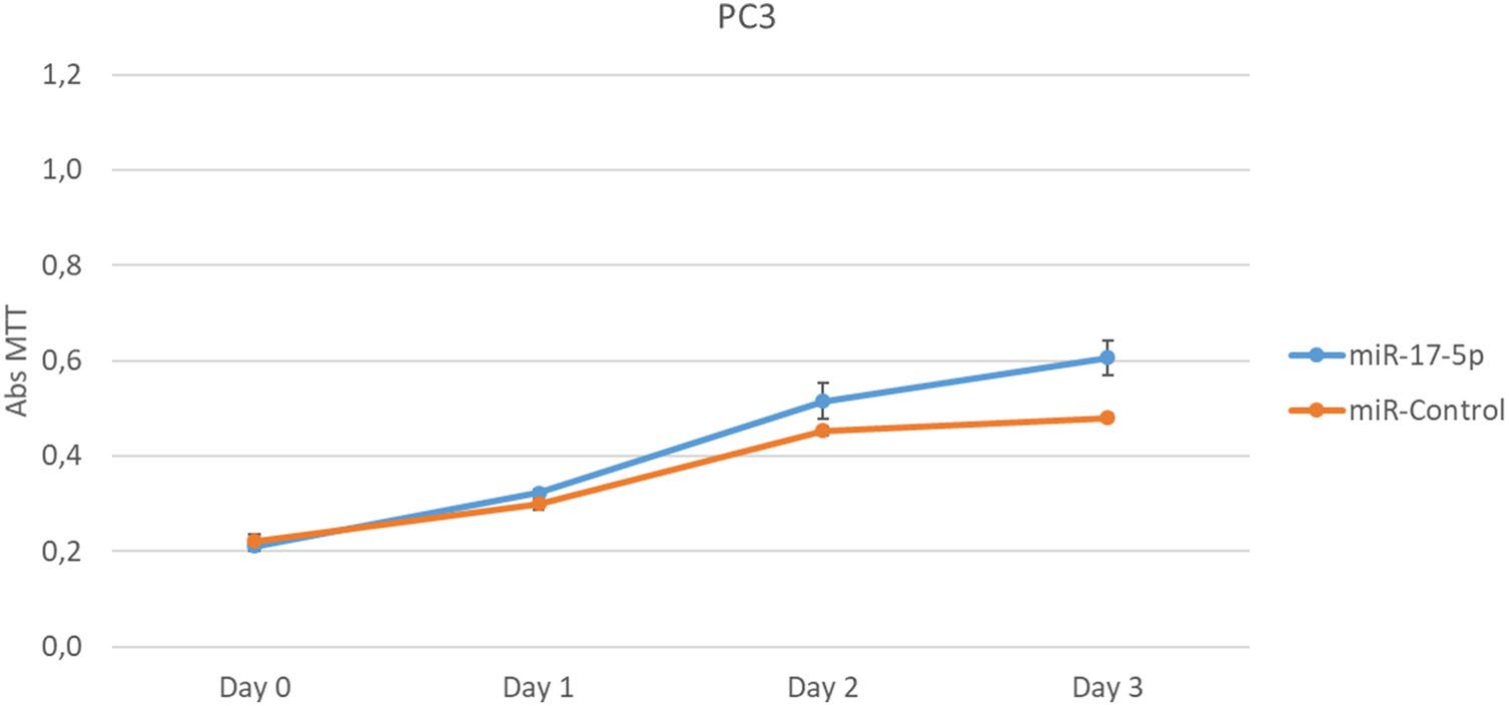
Cell proliferation was measured in PC3 and DU145 cells, and compared with control cells that were not transfected with miR-17-5p. PC3 Cell lines transfected with miR-17-5p showed increased proliferation compared with controls (representative of three experiments), while DU145 cell line did not have significant variation. Results from all experiments are shown in supplementary (Supplementary Figure S2).

PC3 and DU145 cell lines were transfected with miR-17-5p (10 μM) 24 h after being plated. After 24 h of transfection, the cells were treated with MTT over 4 days. The overexpression of miR-17-5p led to proliferation of PC3 cell line, while DU145 cell line did not have significant difference in proliferation compared to controls (non-transfected DU145) (Fig. 4).

### Wound healing analysis

**Figure 5:**
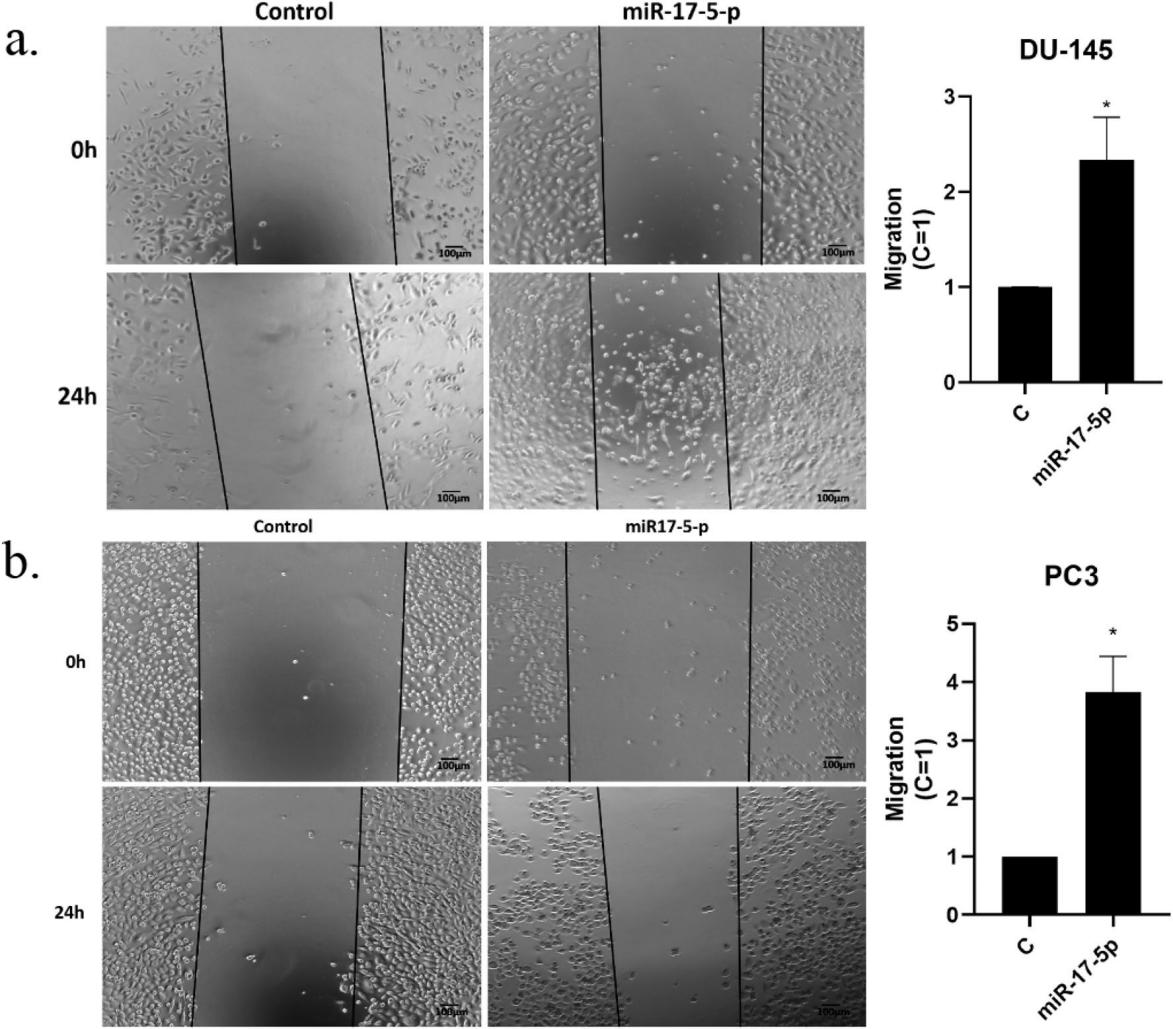
Migration in PC3 and DU145 cell lines were measured using wound healing assays. miR-17-5p transfected cells were compared with controls. Both PC3 and DU145 cell lines showed significant migration (*p* < 0.05) compared to controls. Figure are representative results from three experiments for PC3 and DU145. Full results from the experiments are presented in Supplementary Table S3 (PC3 and DU145). (**a**) Wound healing assays for DU145; (**b**) Wound healing assays for PC3.

The effects of miR-17-5p on PC3 and DU145 cell migration was studied by wound healing analyses. Cells were incubated in serum free culture medium containing a blocking DNA replication mitomycin C (10 µg/L) to avoid cell proliferation. Then the cells were wounded and transfected in the presence of miR-17-5p and absence (control) for 24 h at 37 °C. Photographs of the wound were taken at 0 and 24 h. Areas occupied by migrating cells after 24 h of incubation were calculated by subtracting the background levels at 0 h, and results were plotted (mean ± SEM) in relation to controls (C = 1). miR-17-5p had significantly increased migration at 24 h treatment in PC3 (*p* < 0.05) and DU145 (*p* < 0.05) cell lines, using Student t-test. Representative results from three experiments of wound healing analysis are presented in Fig. 5.

### Invasion analysis

**Figure 6:**
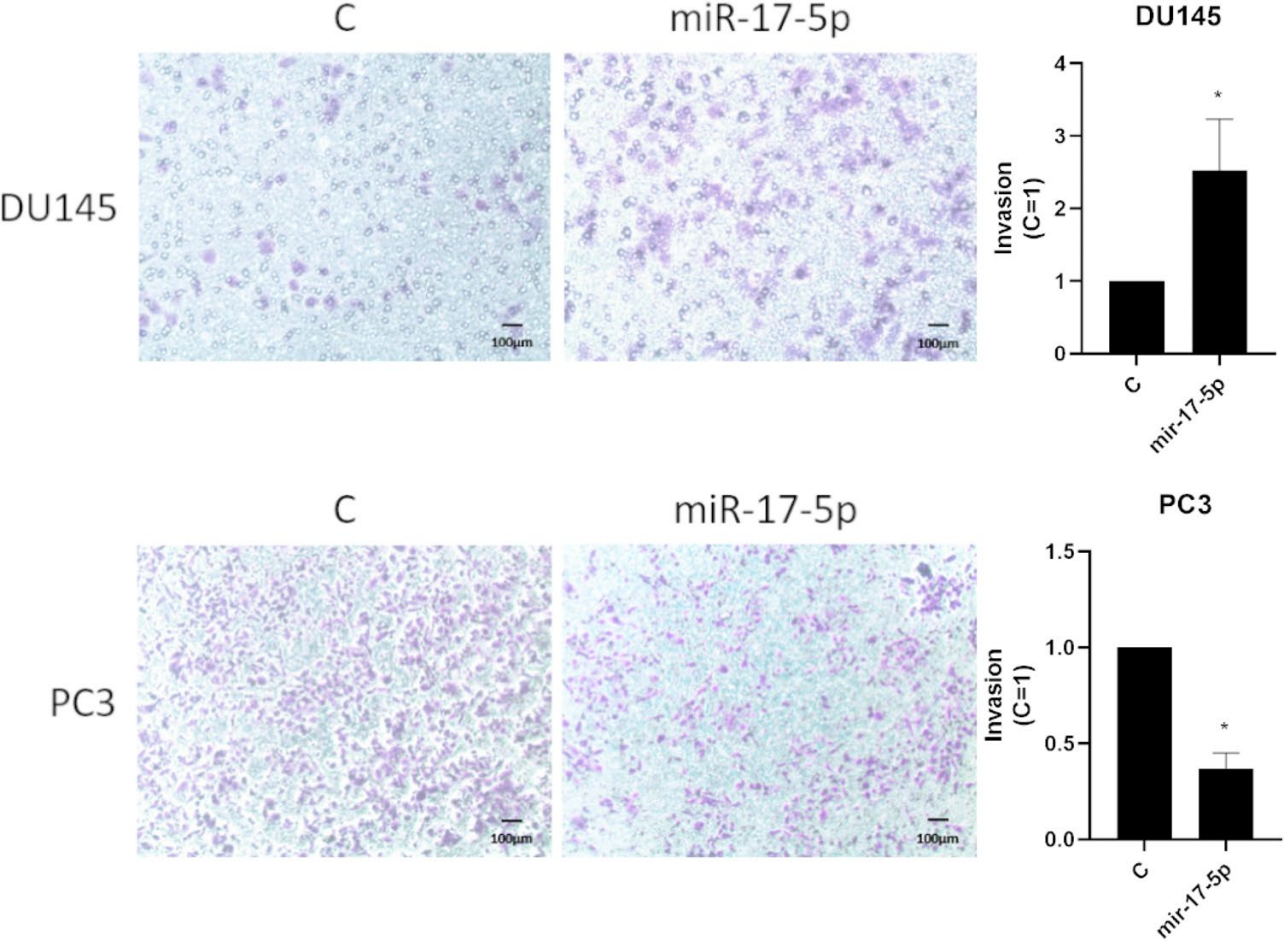
Invasion of PC3 and DU145 cell lines were measured with transfected miR-17-5p compared to controls. DU145 cell line had significant invasion (*p* < 0.05) compared to controls. Figure 6 are representative of three experiments for both DU145 and PC3 cell lines. Full results for all experiments are presented in Supplementary Table S4.

Effects of miR-17-5p transfection on invasion of androgen-independent PCa cell lines was studied using chambers pre-coated with matrigel. Cells were incubated in the absence (control) and presence of miR-17-5p in DU145 and PC3 cell lines (10 nM) at 37 °C for 48 h. Photographs were taken of the membranes containing invaded cells (under the surface of membrane). Images of three random microscope fields were captured and the areas of invaded cells were determined. Results were calculated from three independent experiments in relation to controls (mean ± SEM). miR-17-5p had significantly increased invasion at 48 h treatment in DU145 cell lines (*p* < 0.05, Student t-test). Figure 6 shows representative results from three different experiments of invasion analysis.

## Discussion

In this study, a high expression of miR-17-5p in TE was associated with CF and BF. Additionally, miR-17-5p in TE and in TE + TS were independent prognostic factors for BF. Since the miR-17-5p staining in TE was significant in the multivariate analysis while in TS was not, we chose to focus on TE expression. We did not find an association between PCD and miR-17-5p expression, which may be due to a low number of events in this outcome group. The large number of patients in this cohort, long follow-up time and using ISH to examine both tumor epithelium and tumor stromal areas are some of the strengths to our study. To our knowledge, this is the first ISH study performed on miR-17-5p in PC specimens. The ISH method is adequate for evaluating tissue specific expression considering PCa is a multifocal tumor. However, due to the need for expression in morphologically verified prostate cancer cells we ended up with a large proportion of missing values. This is both a strength as we did not introduce bias like in studies using RT-PCR with non-tissue capturing techniques, and a weakness as small TMA cores yields little tissue available for scoring and could create a systematic bias we are not aware of. Other limitations is the retrospective nature of our study design, as well as we did not pair with healthy tissue controls. Even though the mean follow-up time was 12.4 years, there were relatively few occurrences of CF and PCD. Larger PCa-studies with longer follow-up is needed for more reliable results concerning PCD especially.

Previous prognostic studies on both serum and tissue from PCa patients support our results. However, these studies were all based on RT-PCR^17, 21–23^. Hoey et al*.* discovered that a high serum expression of miR-17-5p was found to be associated with a shorter BFFS in PCa patients after radical prostatectomy by utilizing The Cancer Genome Atlas (TCGA) dataset^17^. An overexpression of the miR-17-92 cluster in PCa tissue is also found associated with shorter time to biochemical recurrence in PCa^21^. Dyson et al*.* found aberrant expression of circulating miR-17 associated with a higher Gleason score (> 6), meaning a more aggressive phenotype^22^. The study did not distinguish between miR-17-5p and -3p^22^. Circular RNA‐mitochondrial tRNA translation optimization 1 expression (Circ-MTO1) inhibits miR-17-5p expression in PCa, and high Circ-MTO1 expression independently predicted favourable overall survival (OS) and disease-free survival (DFS) for PCa patients^23^. Our study was in accordance with previous prognostic studies, finding a worse PCa outcome with higher miR-17-5p expressions.

Meta-analyses with systematic reviews of the prognostic value of miR-17-5p and miR-17-92 cluster in human carcinomas has been published recently^24–30^**.** High miR-17–92 cluster expression was in association with poor OS and DFS in various malignancies^24, 25^. Increased expression of miR-17-5p was also shown to be a significant predictor for poor OS and DFS^26–30^, as well as poor recurrence-free survival (RFS) in different cancers including hepatocellular, colorectal and gastric carcinomas^29, 30^. To our knowledge, there is no published meta-analysis focusing on miR-17-5p in PCa. However, the findings from the existing meta-analyses are in line with the findings in our study, considering we found an association between increased miR-17-5p expression and worse outcome.

miR-17-5p’s function is debated. As a part of miR-17-92 cluster, it is considered to have an oncogenic role in PCa, and to contribute on cancer invasion and metastasis^13, 18^. miR-17-5p acts as an oncomiR in several cancers, among them liver, gastric and CRC^31^. However, in breast-, lung- and prostate cancer, there is conflicting results regarding the role of miR-17-5p^31^. miR-17-5p is often described as an oncomiR in PCa, and the miR is shown overexpressed in PCa cell lines and tissue^15, 16, 21, 32–34^. While other studies reported a downregulation of miR-17-5p in LNCaP cell lines and PCa tissue with a tumor suppressive impact on PCa^35–38^. Dyson et al. reported that both an upregulation and downregulation of circulating miR-17 is associated with aggressive PCa, meaning that miR-17 controls both tumor suppressive and oncogenic genes^22^. This duality is in accordance with the contradictory results on miR-17’s function in PCa progression.

Studies have shown the importance of miR-17-5p in cell proliferation and survival in PCa cell lines^15, 34^. Enhanced expression of miR-17 is shown by Zhang et al*.* to cause cell proliferation and tumor growth in PC3 cells^34^. Yang et al*.* found miR-17-5p to downregulate the cell cycle inhibitor p21, leading to PCa cell proliferation in DU145 cell lines^15^. It is important to note that none of these studies ever mentions the passage of prostate cancer cell lines. With increased number of passages, there was observed different types of changes in phenotype, gene expression, genetic modifications and proteins^39–43^. Lipiec et al*.* observed changes in PC3 response to induction of cell death by radiation after the 47 passage, and they stated that the passage number effect might induce changes resulting in both growth inhibition and apoptosis^44^. The passage number effect could therefore be one potential reason on contradictory reports on cell lines. Our study found that overexpressing miR-17-5p induced the proliferation in PC3 cell lines (Fig. 4), but these results was not significant in DU145 cells (Supplementary Fig. S2). PC3 cell line is from bone metastasis, and DU145 is from brain metastasis and therefore they are cells with different proteins expressions and miR levels. One example of this is the expression of AKT phosphorylated present in PC3^45^ and absent in DU145^46^. This different phenotype could explain why there was proliferation in PC3 through miR-17-5p while in DU145 it was not verified. Further studies are needed to understand the role of miR through the signalling pathways involved with proliferation in PC3 and DU145.

In addition to proliferation, cell migration and invasion are important biological mechanisms pivotal in prostate cancer development. In our study, we found significant migration in miR-17-5p transfected cells compared to controls in PC3 and DU145 cell lines. We also found significant invasion in miR-17-5p transfected DU145 cell lines. miR-17-5p overexpression is shown in other studies to inhibit the tumor suppressors PTEN and TIMP3, causing tumor growth and invasion in PCa cell lines^15, 32^. PTEN is a regulator of the important cell survival PI3K/AKT/mTOR, which is one of the most frequently activated pathways in PCa^15, 47^. TIMP3 it is an inhibitor of metalloproteinases and an extracellular matrix regulator. Silencing TIMP3 with small interfering RNA promoted cell survival and invasion on LNCaP, PC3, DU145 cell lines^15^. If the miR-17-5p overexpression in DU145 and PC3 cells in our experimental model is activating PI3K/AKT pathways or contributing for TIMP3 inhibition, further studies would be needed.

To our knowledge, this study is the largest concerning miR-17-5p expression and clinical outcomes in PCa patients. Our study found miR-17-5p in TE and in TE + TS to be independent prognostic factors for BF in PCa, which have implications for future research on PCa biomarkers. Aberrant expression of miR-17-5p in PCa tissue may have an impact on prognosis, making it an attractive target. miR-expression can be analysed by ISH on routine paraffin-embedded tissue. We suggest miR-17-5p for further validation, which may confirm clinical utility as a prognostic biomarker.

## Conclusion

In this study, we found that a high expression of miR-17-5p in TE and in TE + TS is independently associated with a worse outcome of PCa, as well as a higher risk of developing a more aggressive PCa phenotype. Based on these findings, we propose miR-17-5p as a potential prognostic biomarker in PCa.

## Materials and methods

### Patients

Six-hundred-and-seventy-one consecutive patients who underwent radical prostatectomies at the University Hospital of North Norway (UNN, n = 267), Nordlandssykehuset Bodø (NLSH, n = 63), and Trondheim University Hospital (St. Olav, n = 341) between 1995 and 2005 were collected and clinicopathological variables registered. All patients were diagnosed with adenocarcinoma of the prostate gland. One-hundred-and-thirty-six patients were excluded due to missing tissue blocks (n = 130), insufficient follow-up (n = 1), other cancer within five years of PCa diagnosis (n = 4) and pelvic radiation or hormonal therapy prior to radical prostatectomy (n = 1), leaving sufficient data from 535 patients. Clinicopathological variables, such as age at surgery, preoperative PSA level and surgical procedures, were collected for all patients. Different outcomes were registered until the last follow-up in December 31st, 2015, and the median follow-up for survivors was 12.4 years (range 1.5–20 years). The median follow-up of survivors and the outcomes biochemical failure (BF) was 9.8 years (range 0.15–18.8 years), clinical failure (CF) was 11.3 years (range 0.15–19.2 years) and prostate cancer death (PCD) was 12.3 years (range 0.15–20.4 years). BF was defined as a PSA value ≥ 0.4 ng/ml, and CF was defined as radiologically verified metastasis or a recurring palpable tumor. BF-free survival (BFFS), CF-free survival (CFFS) and PCD-free survival (PCDFS) was calculated as time from surgery to either last follow up date or date where the event occurs. For more information about the prostate cancer cohort see previously published articles (35). In 2018, the tumors were re-evaluated and graded by a skilled uro-pathologist (ER), and this was done in accordance with the new WHO guidelines^48, 49^.

### Tissue microarray construction (TMAs)

This study used twelve TMA blocks consisting of cores from formalin-fixed paraffin-embedded (FFPE) tissue blocks. One uro-pathologist (ER) selected the most representative areas of neoplastic cells (tumor epithelium) and tumor associated stromal areas (tumor stroma) for sampling. Samples from the areas were collected using a 0.6 mm needle, and the cores were then inserted into paraffin blocks. A tissue-arraying device (Beecher Instruments, Silver Springs, MD, USA) was used to construct the TMAs. Detailed information about this method has previously been reported^50^.

### In situ hybridization (ISH)

The Ventana Discovery Ultra instrument (Ventana Medical Inc, Arizona, USA) was used for the chromogenic ISH. The buffers and the detection reagents were purchased from Roche (Basel, Switzerland). The miRCURY LNA detection probe hsa-miR-17-5p, (No. 619852-360), the positive control (U6 hsa, No. 160010126), and negative control (scrambled-miRNA, No. 157057117) was purchased from Exiqon (Vedbaek, Denmark). The water, buffers and equipment used were all RNAse-free in order to prevent RNA degradation. A TMA multi-organ block was utilized to test both unmasking pre-treatments and probe concentrations in order to optimize the detection method, and both normal and malignant tissue was studied on control TMA blocks to validate the staining. The temperatures for hybridization and stringent wash were tested and optimized with recommended temperatures as guidelines. A U6 snRNA control probe at 1.5 nM concentration was used to secure the sensitivity level of the ISH method. Optimal sensitivity was judged as bright nuclear signal at concentrations between 0.1–2.0 nM for U6. Furthermore, by visualizing powerful nuclear staining with a light microscope, the U6 indicated a low amount of RNA degradation. The concentrations of the other probes were 10 nM for negative control (scrambled-miRNA) and 20 nM for miR-17-5p. The concentration of every miR-probe was optimized by testing at different concentrations, and choosing those, which gave clear staining of the TMA cores without unspecific positive background staining. Exiqon, the supplier of the detection probes, recommend concentrations in the range of 20–80 nM for their miR-probes.

### The ISH procedure

The 4 µm thick TMA sections were incubated at 60 °C to dry and dispose of excess paraffin. Liquid Coverslip oil (Roche, 5264839001) was added to prevent slides from drying and to ensure correct incubation throughout every slide. The deparafinization was performed in cycles at 68 °C (3 × 12 min). To remove the formalin fixations cross linking effects, the slides underwent target unmasking at 95 °C with CC1 buffer (Roche, 6414575001) for 40 min. The different probes were added manually, and then followed the denaturation and hybridization. The denaturation was performed with 8 min incubation at 90 °C. The hybridization was performed at different temperatures for the different probes; 54 °C for miR-17-5p, 55 °C for U6 and 57 °C for scramble miR. Here the slides were incubated for 60 min. 2.0X RiboWash was used for the stringent washes (2 × 8 min), and SSPE buffer was used at the same temperatures as the hybridization for each probe. For blocking unspecific bindings an antibody block (Roche, 5268869001) was used at 37 °C for 16 min. For immunologic detection an alkaline phosphatase (AP)-conjugated anti DIG (Anti-DIG-AP multimer, Roche 07256302001) was incubated at 37 °C for 20 min. In order to detect the miRNAs, the slides were rinsed and NBT/BCIP (ChromoMap Blue kit, Roche, 526661001) was added to substrate enzymatic reactions at 37 °C for 60 min. Sections were then again rinsed, and Red Stain II (Roche, 5272017001) was used to counterstain for 4 min. The slides were washed by hand in tap water, dehydration was performed with gradients of ethanol solutions to Xylene, and lastly Histokitt mounting medium was used to arrange the slides.

### Cell culture

The functional properties of miR-17-5p were evaluated in two different prostate cancer cell lines: The human androgen-independent prostate cancer cell lines DU145 (ATCC HTB-81) derived from brain metastasis and PC3 (ATCC CRL-1435) derived from bone metastasis. We used PC3 in all experiments below passage 46 and DU145 below 69. These cells (2 × 10^5^ cells/ml) were cultured in Opti-MEM I (1x) medium without phenol red (cat.# 11058-021, GIBCO, RF, UK), supplemented with 5% of fetal bovine serum (cat.# S0415, Biochrom, Berlin, Germany) and Penicillin Streptomycin 1% (cat.# 15140-148, Gibco, NY, USA), in a humidified atmosphere with 5% CO_2_:95% air, at 37 °C, for 72 h. Afterwards, the culture medium was replaced by one without serum 24 h before the experiments. At this stage, the cells were 85%–90% confluent.

### Viability assay

For the colorimetric proliferation assay, 5 × 10^3^ cells/well (DU145) and 3 × 10^3^ cells/well (PC3) were cultured in 96-well plates. At different time points, cells were incubated with 12 mM of [3-(4,5-dimethylthiazol-2-yl)-2,5-diphenyltetrazolium bromide] (MTT, 5 mg/ml) (cat.# M6494, Invitrogen, OR, USA). For 4 h at 37 °C. The formazan crystals produced were solubilized by addition of 0,01 M HCl/SDS (cat.# 28,312, Thermo Scientific, IL, USA) and mixed thoroughly with the pipette. The cells were incubated at 37 °C overnight to dissolve the formazan and the absorbance was measured in the CLARIOstar plate reader (BMG Labtech, Ortenberg, Germany) at 570 nm.

### Cell transfection

Cells were transiently transfected with either 10 µM has-miR-17-5p Pre-miR miRNA Precursor (catalog# PM12412, Thermo Fisher Scientific, USA), alongside the Cy3 Dye-Labeled Pre-miR Negative Control #1 (catalog# AM17120, Thermo Fisher Scientific, USA) using the transfection reagent Lipofectamine RNAiMAX (catalog#13778075, Thermo Fisher Scientific, USA). Transfected Cy3 Dye-Labeled Pre-miR Negative Control emits fluorescent light when exposed to UV-light, and using a fluorescence microscope, the transfection efficiency was evaluated to be 80–95%.

### Wound healing analysis

miR-17-5p transfection in PC3 and DU145 PCa cell lines. 2 × 10^5^ cells/well were grown in a 24-well plate for the wound scratch assay. PC3 and DU145 cells were washed using PBS. In order to avoid cell proliferation the cells were incubated in serum free culture medium containing the blocking DNA replication mitomycin C (10 µg/L). By using 200 µl sterile pipette tips the cells were wounded and then washed to remove detached cells and debris^51^. After 4 h the cells were transfected with Cy3 Dye-Labeled Pre-miR Negative Control #1 (catalog# AM17120, Thermo Fisher Scientific, USA) (control, basal level of cellular function) or has-miR-17-5p Pre-miR miRNA Precursor (catalog# PM12412, Thermo Fisher Scientific, USA) at 37 °C for 24 h. Photographs of the same areas of the wound were taken at 0 and 24 h to measure the wound-closure in controls and in cells transfected with miR-17-5p^51^. Nikon Eclipse TS100 inverted optical microscope was used to take images, and it was analyzed by Micrometrics SE Premium 4 software. By subtracting the background levels at 0 h, the areas occupied by migrating cells after 24 h of incubation (control and transfected cells) was calculated. The results were plotted (mean ± SEM) in relation to control (C = 1).

### Invasion analysis

PC3 and DU145 cells (2 × 10^5^ cells) in serum free culture medium were seeded in ThincertR chambers (Greiner Bio-one, Kremsmünster, Austria) with polyethylene terephthalate membranes, 8 mm in pore size, pre-coated with 50 mL of phenol red-free Matrigel (Gibco). The chambers were put in a 24-well plates with culture medium (5% FBS in the lower chamber)^51, 52^. PC3 and DU145 cells in upper chambers were incubated with Cy3 Dye-Labeled Pre-miR Negative Control #1 (catalog# AM17120, Thermo Fisher Scientific, USA) (control, basal level of cellular function) or has-miR-17-5p Pre-miR miRNA Precursor (catalog# PM12412, Thermo Fisher Scientific, USA) (10 μM) for 48 h at 37 °C. Cell invasion analyses were performed as previously described^51^. 10 mM PBS was used to wash the chambers thoroughly, then fixed in 4% paraformaldehyde for 30 min, and stained with 0.2% crystal violet for 10 min. A cotton swab was used to remove the non-invading cells from the membrane upper surface. Then photographs were taken of the membranes containing the invaded cells (under the surface of membrane). Images were captured in duplicate of three random microscope fields, using an inverted optical microscope Nikon Eclipse TS100. Image J software was used to determine the areas of cell invasion. Results were plotted (mean ± SEM) in relation to control (C = 1).

### Statistical methods

IBM SPSS version 26 (IBM, SPSS inc., Chicago, IL, USA) was used for all statistical analyses. One pathologist (ER) and a trained investigator (LMI/MJS) scored the expression of miR-17-5p semi-quantitatively, and a two-way random effects model with absolute agreement was used to test inter-observer reliability. Pearson’s Chi-square analysis and Fisher's exact test was used to explore relationships between miR-17-5p (dichotomized) and clinicopathological variables. For the univariate survival analysis, the Kaplan–Meier method was used to draw survival curves and the log-rank test was used to measure the statistical significance between them. At 192 months, less than 10% of the patients were at risk and the curves were concluded. Cox regression models (backward conditional) were utilized for multivariate analyses. Only outcomes with a *p* value ≤ 0.05 from the univariate analysis were included. The probability for step-wise entry and removal was 0.05 and 0.10. The threshold p-value for statistical significance in all analyses was *p* < 0.05.

### Ethics

Regional Committees for Medical and Health Research Ethics (REK North, Ref. no: 2009/1393) has approved this study. The compulsory re-approvals were also completed in 2016 and 2019. Written consent was considered redundant by REK North because of the retrospective study design, since many of the patients were deceased (average age in 2005: 67 y/o) and most of the material was collected more than a decade ago (tissue collected from 1998 to 2005). Informed consent was waived by the Regional Committees for Medical and Health Research Ethics (REK North) for the entire study. All patients were de-identified using trial numbers before scoring and statistical analyses. The only purpose of linking trial numbers with patients was for collecting clinical information. The REMARK recommendations were followed with regards of the reporting and analysis of data, and in the reporting of the clinicopathological information and biomarker expression^53^. Data aggregation and storage of the database was approved by The Norwegian Centre for Research Data (NSD). We hereby confirm that all research was performed in accordance with relevant guidelines/regulations.

## Supplementary Information

Supplementary Information.

## Data Availability

The datasets generated during and/or analysed during the current study are available from the corresponding author on reasonable request.

## References

[1] Ferlay J (2015). Cancer incidence and mortality worldwide: sources, methods and major patterns in GLOBOCAN 2012. Int. J. Cancer.

[2] Rawla P (2019). Epidemiology of prostate cancer. World J. Oncol..

[3] Sharma N, Baruah MM (2019). The microRNA signatures: aberrantly expressed miRNAs in prostate cancer. Clin. Transl. Oncol..

[4] Weidle UH, Epp A, Birzele F, Brinkmann U (2019). The functional role of prostate cancer metastasis-related micro-RNAs. Cancer Genom. Proteom..

[5] MicroRNA Target Identification (2019). Methods and Protocols.

[6] Fan R (2019). Small molecules with big roles in microRNA chemical biology and microRNA-targeted therapeutics. RNA Biol..

[7] Razdan A, de Souza P, Roberts TL (2018). Role of MicroRNAs in treatment response in prostate cancer. Curr. Cancer Drug Targets.

[8] Balacescu, O., Dumitrescu, R. G. & Marian, C. in *Cancer Epigenetics for Precision Medicine : Methods and Protocols* (eds Ramona G. Dumitrescu & Mukesh Verma) 103–117 (Springer New York, 2018).

[9] Mitchell PS (2008). Circulating microRNAs as stable blood-based markers for cancer detection. Proc. Natl. Acad. Sci. USA.

[10] Chen X (2008). Characterization of microRNAs in serum: a novel class of biomarkers for diagnosis of cancer and other diseases. Cell Res..

[11] Bai X, Hua S, Zhang J, Xu S (2019). The MicroRNA family both in normal development and in different diseases: the miR-17-92 cluster. Biomed. Res. Int..

[12] Khuu C, Utheim TP, Sehic A (2016). The three paralogous MicroRNA clusters in development and disease, miR-17-92, miR-106a-363, and miR-106b-25. Scientifica (Cairo).

[13] Liu H (2019). The SOX4/miR-17-92/RB1 axis promotes prostate cancer progression. Neoplasia.

[14] Dellago H, Bobbili MR, Grillari J (2017). MicroRNA-17-5p: at the crossroads of cancer and aging - a mini-review. Gerontology.

[15] Yang X (2013). Both mature miR-17-5p and passenger strand miR-17-3p target TIMP3 and induce prostate tumor growth and invasion. Nucleic Acids Res..

[16] Volinia S (2006). A microRNA expression signature of human solid tumors defines cancer gene targets. Proc. Natl. Acad. Sci. USA.

[17] Hoey C (2019). Circulating miRNAs as non-invasive biomarkers to predict aggressive prostate cancer after radical prostatectomy. J. Transl. Med..

[18] Zhou P (2016). miR-17-92 plays an oncogenic role and conveys chemo-resistance to cisplatin in human prostate cancer cells. Int. J. Oncol..

[19] Melbo-Jorgensen C (2014). Stromal expression of MiR-21 predicts biochemical failure in prostate cancer patients with Gleason score 6. PLoS ONE.

[20] Moi L, Braaten T, Al-Shibli K, Lund E, Busund LR (2019). Differential expression of the miR-17-92 cluster and miR-17 family in breast cancer according to tumor type; results from the Norwegian Women and Cancer (NOWAC) study. J. Transl. Med..

[21] Feng S, Qian X, Li H, Zhang X (2017). Combinations of elevated tissue miRNA-17-92 cluster expression and serum prostate-specific antigen as potential diagnostic biomarkers for prostate cancer. Oncol. Lett..

[22] Dyson G (2018). The extrema of circulating miR-17 are identified as biomarkers for aggressive prostate cancer. Am. J. Cancer Res..

[23] Hu Y, Guo B (2020). Circ-MTO1 correlates with favorable prognosis and inhibits cell proliferation, invasion as well as miR-17-5p expression in prostate cancer. J. Clin. Lab Anal..

[24] Liu F (2017). Prognostic role of miR-17-92 family in human cancers: evaluation of multiple prognostic outcomes. Oncotarget.

[25] Zhang K (2017). Prognostic value of high-expression of miR-17-92 cluster in various tumors: evidence from a meta-analysis. Sci. Rep..

[26] Duan F (2018). Quantifying the prognostic significance of microRNA-17/17-5P in cancers: a meta-analysis based on published studies. Cancer Manag. Res..

[27] Wang Z (2018). Prognostic value of miR-17-5 p in gastrointestinal cancers: a systematic review and meta-analysis. Onco Targets Ther..

[28] Zheng Q, Chen C, Guan H, Kang W, Yu C (2017). Prognostic role of microRNAs in human gastrointestinal cancer: A systematic review and meta-analysis. Oncotarget.

[29] Kong W (2018). Prognostic value of miR-17-5p in cancers: a meta-analysis. Onco Targets Ther..

[30] Huang C, Yu M, Yao X (2018). MicroRNA-17 and the prognosis of human carcinomas: a systematic review and meta-analysis. BMJ Open.

[31] Bobbili MR, Mader RM, Grillari J, Dellago H (2017). OncomiR-17-5p: alarm signal in cancer?. Oncotarget.

[32] Dhar S, Kumar A, Rimando AM, Zhang X, Levenson AS (2015). Resveratrol and pterostilbene epigenetically restore PTEN expression by targeting oncomiRs of the miR-17 family in prostate cancer. Oncotarget.

[33] Wang X, Wang R, Wu Z, Bai P (2019). Circular RNA ITCH suppressed prostate cancer progression by increasing HOXB13 expression via spongy miR-17-5p. Cancer Cell Int..

[34] Zhang C (2014). ROCK has a crucial role in regulating prostate tumor growth through interaction with c-Myc. Oncogene.

[35] Gong AY (2012). miR-17-5p targets the p300/CBP-associated factor and modulates androgen receptor transcriptional activity in cultured prostate cancer cells. BMC Cancer.

[36] Dai H (2018). MiR-17 regulates prostate cancer cell proliferation and apoptosis through inhibiting JAK-STAT3 signaling pathway. Cancer Biother. Radiopharm..

[37] Ottman R, Levy J, Grizzle WE, Chakrabarti R (2016). The other face of miR-17-92a cluster, exhibiting tumor suppressor effects in prostate cancer. Oncotarget.

[38] Dankert JT (2018). The deregulation of miR-17/CCND1 axis during neuroendocrine transdifferentiation of LNCaP prostate cancer cells. PLoS ONE.

[39] Esquenet M, Swinnen JV, Heyns W, Verhoeven G (1997). LNCaP prostatic adenocarcinoma cells derived from low and high passage numbers display divergent responses not only to androgens but also to retinoids. J. Steroid Biochem. Mol. Biol..

[40] Chang-Liu CM, Woloschak GE (1997). Effect of passage number on cellular response to DNA-damaging agents: cell survival and gene expression. Cancer Lett..

[41] Briske-Anderson MJ, Finley JW, Newman SM (1997). The influence of culture time and passage number on the morphological and physiological development of Caco-2 cells. Proc. Soc. Exp. Biol. Med..

[42] Wenger SL (2005). Comparison of established cell lines at different passages by karyotype and comparative genomic hybridization. Biosci. Rep..

[43] Sambuy Y (2005). The Caco-2 cell line as a model of the intestinal barrier: influence of cell and culture-related factors on Caco-2 cell functional characteristics. Cell Biol. Toxicol..

[44] Lipiec EW (2012). Changes in cellular response to the damage induced in PC-3 prostate cancer cells by proton microbeam irradiation. Gen. Physiol. Biophys..

[45] Lombardi AP (2016). Estrogen receptor beta (ERβ) mediates expression of β-catenin and proliferation in prostate cancer cell line PC-3. Mol. Cell Endocrinol..

[46] Souza DS (2019). Estrogen receptors localization and signaling pathways in DU-145 human prostate cancer cells. Mol. Cell Endocrinol..

[47] Carver BS (2011). Reciprocal feedback regulation of PI3K and androgen receptor signaling in PTEN-deficient prostate cancer. Cancer Cell.

[48] Epstein, J. I. *et al.* The 2014 International Society of Urological Pathology (ISUP) Consensus Conference on Gleason Grading of Prostatic Carcinoma: Definition of Grading Patterns and Proposal for a New Grading System. *Am J Surg Pathol* **40**, 244–252, 10.1097/PAS.0000000000000530 (2016). 10.1097/PAS.0000000000000530 26492179

[49] Epstein JI (2016). A contemporary prostate cancer grading system: a validated alternative to the Gleason Score. Eur. Urol..

[50] Bremnes RM (2002). High-throughput tissue microarray analysis used to evaluate biology and prognostic significance of the E-cadherin pathway in non-small-cell lung cancer. J. Clin. Oncol..

[51] Lombardi, A. P. G., Vicente, C. M. & Porto, C. S. Estrogen Receptors Promote Migration, Invasion and Colony Formation of the Androgen-Independent Prostate Cancer Cells PC-3 Through β-Catenin Pathway. *Front Endocrinol (Lausanne)* **11**, 184,10.3389/fendo.2020.00184 (2020). 10.3389/fendo.2020.00184 PMC7160699 32328032

[52] Vicente CM, Lima MA, Nader HB, Toma L (2015). SULF2 overexpression positively regulates tumorigenicity of human prostate cancer cells. J. Exp. Clin. Cancer Res..

[53] McShane LM (2005). REporting recommendations for tumour MARKer prognostic studies (REMARK). Br. J. Cancer.

