# Supplementary material for: High expression of miR-17-5p in tumor epithelium is a predictor for poor prognosis for prostate cancer patients
Source: Sci Rep. 2021 Jul 5;11:13864. doi: 10.1038/s41598-021-93208-6 (PMC8257715; doi:10.1038/s41598-021-93208-6)
Supplement: Supplementary file 1 — Supplementary Information. [file 41598_2021_93208_MOESM1_ESM.pdf]

High expression of miR-17-5p in tumor epithelium is a predictor for poor prognosis for prostate cancer patients

Maria Jenvin Stoen, PhD-student[1]
Sigve Andersen, PhD, M.D[2,4]
Mehrdad Rakaee, PhD, MSc[1,2]
Mona Irene Pedersen, Head engineer[2]
Lise Martine Ingebriktsen, PhD-student, MSc[1,5]
Roy M. Bremnes, PhD, M.D[2,4],
Tom Donnem, PhD, M.D[2,4],
Ana Paola Giometti Lombardi, Head engineer[1]
Thomas Karsten Kilvaer, PhD, M.D[1,4]
Lill-Tove Rasmussen Busund, PhD, M.D[1,3]
Elin Richardsen, PhD, M.D[1,3]

[1]Translational Cancer Research Group, Institute of Medical Biology, UiT The Arctic University of Norway, Tromso, North Norway.
[2]Translational Cancer Research Group, Institute of Clinical Medicine, UiT The Arctic University of Norway, Tromso, North Norway.
[3]Department of Clinical Pathology, University Hospital of North Norway, Tromso, Norway.
[4]Department of Oncology, University Hospital of North Norway, Tromso, North Norway.
[5]Centre for Cancer Biomarkers CCBIO, Department of Clinical Medicine, Section for Pathology, University of Bergen, Bergen, N-5021, Norway.

*Corresponding author:

Maria Jenvin Stoen
Translational Cancer Research Group
Institute of Medical Biology
UiT The Arctic University of Norway
9037 Tromso, Norway
Telephone +47 97419736
Fax: +47 77627204
E-mail: mst207@post.uit.no

Supplementary Table S1

Characteristic	Patients		BF (n = 200)		CF (n = 56)		PCD (n = 18)	
	n	%	5-year EFS (%)	p	10-year EFS (%)	p	10-year EFS (%)	p
**Age**				0.237		**0.038**		0.404
≤ 65	357	67	77		94		98	
> 65	178	33	70		91		98	
**ISUP Grade**				**<0.001**		**<0.001**		**<0.001**
1	183	34	83		98		99	
2	219	41	77		94		99	
3	81	15	70		90		96	
4	17	3	58		86		94	
5	35	7	37		65		90	
**Preop. PSA**				**<0.001**		**0.029**		**0.003**
PSA < 10	308	58	81		95		99	
PSA > 10	221	41	68		89		97	
Missing	6	1						
**Tumor size**				**<0.001**		**0.002**		0.085
< 20	250	47	82		96		99	
≥ 20	285	53	68		90		97	
**pT-stage**				**<0.001**		**<0.001**		**0.001**
pT2	374	70	83		97		99	
pT3a	114	21	61		87		98	
pT3b	47	9	43		74		91	
**pN-stage**				**<0.001**		**<0.001**		**<0.001**
NX	264	49	79		96		99	
N0	268	50	72		90		97	
N1	3	1	0		33		67	
**PNI**				**<0.001**		**<0.001**		**<0.001**
Yes	134	25	60		83		95	
No	401	75	80		96		99	
**LVI**				**<0.001**		**<0.001**		**<0.001**
Yes	43	8	47		69		89	
No	492	92	77		95		99	
**Margin**				**0.049**		0.198		0.843
Not free	286	53	69		96		98	
Free	249	47	81		90		98	
**PAM**				0.063		0.427		0.128
Yes	210	39	77		93		98	
No	325	61	74		92		98	
**PCM**				**<0.001**		**<0.001**		**0.022**
Yes	154	29	57		85		96	
No	381	71	82		96		99	
**Surg. method**				0.466		0.308		0.965

Perineal	100	19	67		95		99	
Retropubic	435	81	77		92		98	

Clinicopathological variables as predictors of biochemical failure, clinical failure and death of prostate cancer. (Univariate analysis; log-rank test, N = 535.) Significant p-values in bold. Abbreviations: BF = biochemical failure; CF = clinical failure; EFS = event-free survival; ISUP Grade = ISUP Grade Groups; LVI = lympho-vascular infiltration; p = p-value; PAM = positive apical margin; PCD = prostate cancer death; PCM = positive circumferent margin; PNI = perineural infiltration; Preop = preoperative; PSA = prostate specific antigen; Surg. method = Surgical method.

Supplementary Table S2

**Characteristic**	**miR-17-5p**		
	**TE (p)**	**TS (p)**	**TE + TS (p)**
PAM	0.022	**0.001**	**0.004**
Perineural infiltration	**< 0.001**	**< 0.001**	**< 0.001**
Vascular infiltration	NS	0.015	NS
pT-stage: 2, 3a or 3b	NS	0.026	0.030
Type of prostatectomy	NS	0.039	0.014

Chi-square analyses for relationships between median scores of miR-17-5p in TE, TS and TE+TS, compared to clinicopathological variables. P-values < 0.01 in bold. Abbreviations: NS = not significant; p = p-value; PAM = positive apical margin; TE = tumor epithelium; TS = tumor stroma.

Supplementary Table S3

	DU145		PC3	
**Treatment**	**Absolute Migration (Average)**	**Graphic (C=1)**	**Absolute Migration (Average)**	**Graphic (C = 1)**
**Exp 1**				
**C**	146,51µm	1	47,41µm	1
**miR-17-5p**	251,73µm	1,71	221µm	4,66
**Exp 2**				
**C**	88,32µm	1	69,92µm	1
**miR-17-5p**	220,90µm	2,5	223,34µm	3,20
**Exp 3**				
**C**	155µm	1	27,83µm	1
**miR-17-5p**	453,05µm	2,79	100,84µm	3,62

Wound Healing Analysis showing the effects of transfected miR-17-5p on PC3 and DU145 cell migration. PC3 and DU145 cell lines showed significant migration ($p < 0.05$) compared to controls, by Student t-test. Abbreviations: C = Control; Exp = experiment.

Supplementary Table S4

	DU145		PC3	
**Treatment**	**Invasion (Average)**	**Graphic (C=1)**	**Invasion (Average)**	**Graphic (C = 1)**
**Exp 1**				
**C**	49 cells	1	856 cells	1
**miR-17-5p**	76 cells	1,55	228 cells	0,26
**Exp 2**				
**C**	10 cells	1	854 cells	1
**miR-17-5p**	30 cells	3,15	405 cells	0,47
**Exp 3**				
**C**	12 cells	1	596 cells	1
**miR-17-5p**	33 cells	2,89	226 cells	0,37

Invasion assays showing the effects of transfected miR-17-5p on PC3 and DU145 cell invasion. DU145 cell line showed significant invasion ($p < 0.05$, Student t-test) compared to controls.

Abbreviations: C = Control; Exp = experiment.

Supplementary Figure S1

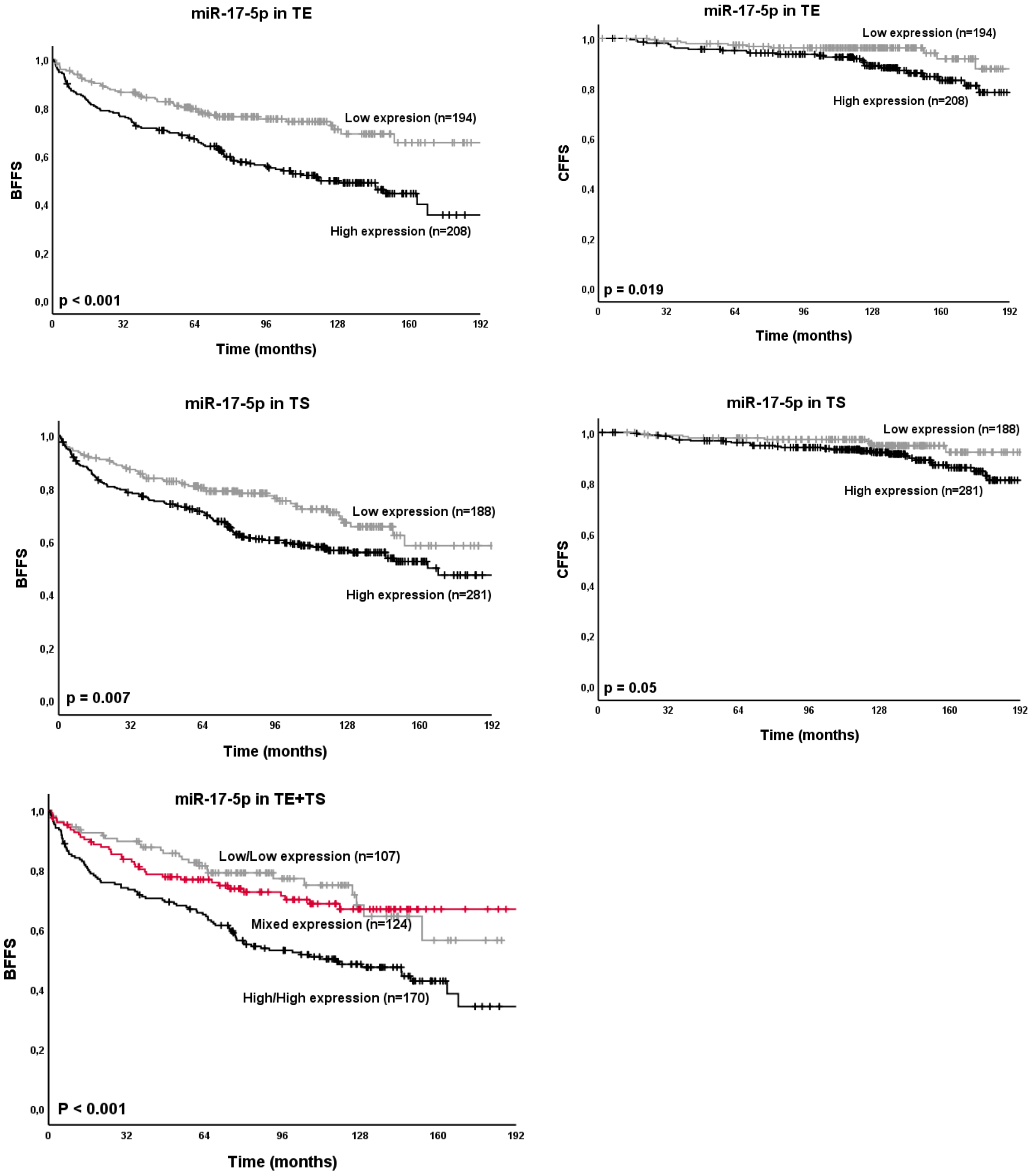

Kaplan-Meier survival curves presenting miR-17-5p in TE, TS and TE+TS with the prostate cancer outcome variables BF and CF. miR-17-5p in TE and TS was dichotomized into low and high expression. TE+TS was created by the sum of the dichotomized TE and TS variables, creating 3 categories; 0 (low/low expression), 1 (mixed expression) and 2 (high/high expression). Abbreviations: BFFS = biochemical failure-free survival; CFFS = clinical failure-free survival; p = p-value; TE = tumor epithelium; TS = tumor stroma.

Supplementary Figure S2

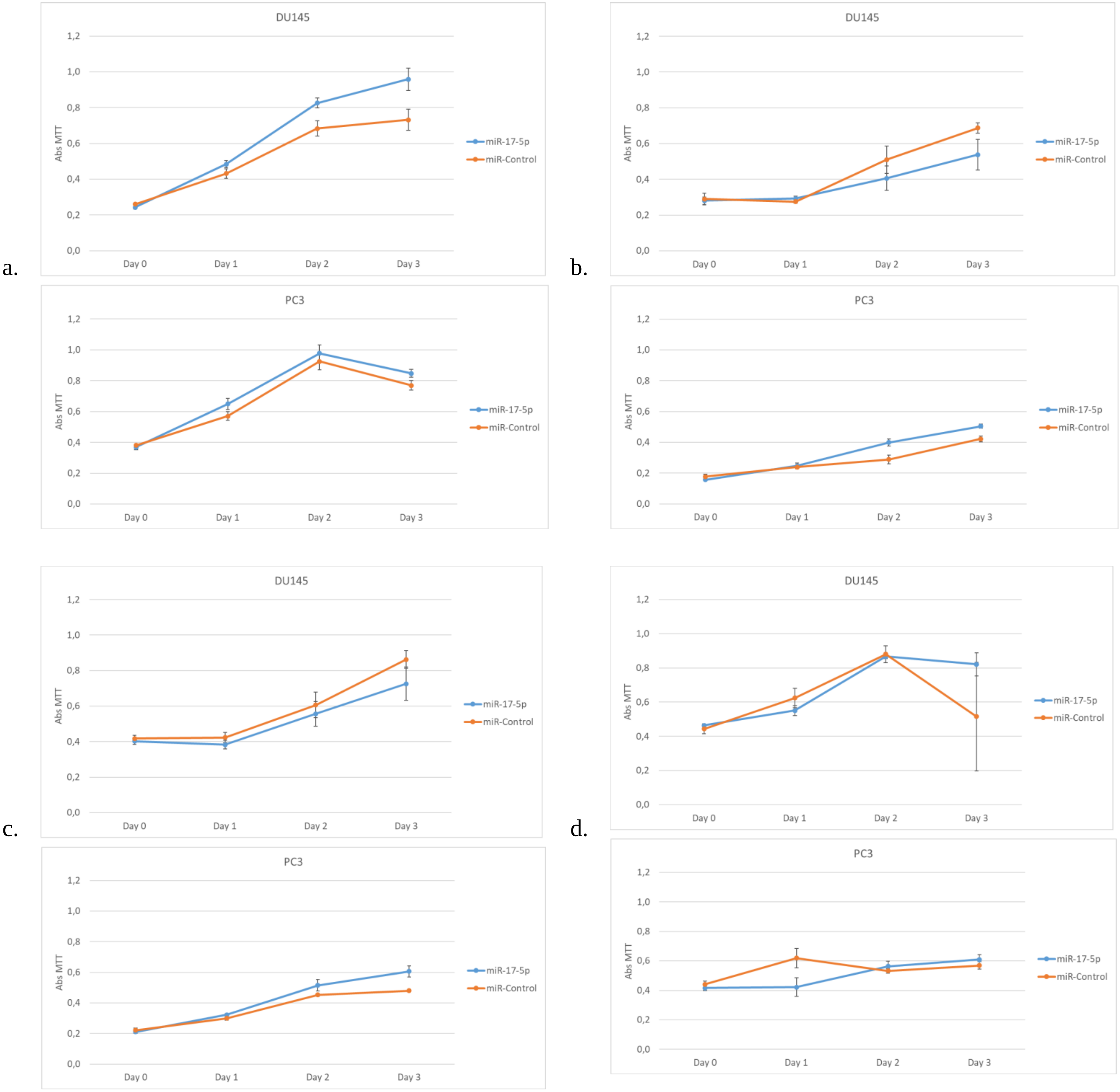

Figure showing all cell line results from proliferation study. Cell proliferation was measured in PC3 and DU145 cell lines, and compared with controls. (a and b) Significant results from proliferation study in both DU145 and PC3 cell lines; (c) Significant results in PC3 cell lines, but not in DU145; (d) Results from both PC3 and DU145 cell lines were not significant.
